# Supplementary material for: Multimorbidity combinations, costs of hospital care and potentially preventable emergency admissions in England: A cohort study
Source: PLoS Med. 2021 Jan 13;18(1):e1003514. doi: 10.1371/journal.pmed.1003514 (PMC7815339; doi:10.1371/journal.pmed.1003514)
Supplement: S2 Appendix — (DOCX) [file pmed.1003514.s002.docx]

# S2 Appendix. Multimorbidity ICD-10 codes

(Table adapted from Table 1 in Tonelli, M., Wiebe, N., Fortin, M., Guthrie, B., Hemmelgarn, B.R., James, M.T., Klarenbach, S.W., Lewanczuk, R., Manns, B.J., Ronksley, P. and Sargious, P., 2015. Methods for identifying 30 chronic conditions: application to administrative data. *BMC medical informatics and decision making*, *15*(1), p.31.)

| **Morbidity** | **ICD-10** | **Included in NHS compulsory comorbidity coding? (**<https://hscic.kahootz.com/gf2.ti/f/762498/27838341.1/PDF/-/Coding_Clinic_032017_V6.2.pdf>)  “When other conditions, not contained within the comorbidity list, have been identified by the responsible consultant as clinically relevant, these conditions must also be coded.” |
| --- | --- | --- |
| Alcohol misuse | E52, F10, G62.1, I42.6,K29.2, K70.0, K70.3, K70.9, T51, Z50.2, Z71.4, Z72.1 | Yes |
| Asthma | J45 | Yes |
| Atrial fibrillation | I48.0 | No |
| Cancer, lymphoma | C81–C85, C88, C90.0, C90.2, C96 | No (but, “Typically a cancer centre uses a continuous medical record for a patient admitted for a course of chemotherapy to treat malignant neoplasm. The co-morbidities will be recorded in the medical record on the first admission but will not always be repeated”) |
| Cancer, metastatic | C77–C80 |  |
| Cancer, non-metastatic (breast, cervical, colorectal, lung, prostate) | C18-C21, C33-C34, C38.4, C45.0, C46.71, C50, C53, C61, D01.0-D01.3, D02.2, D05-D06, D07.5 |  |
| Chronic heart failure | I09.9, I25.5, I42.0, I42.5–I42.9, I43, I50 | Yes |
| Chronic kidney disease | N00-N23 | Yes |
| Chronic pain  (2 hospitalisations) | F45.4, M08.1, M25.50, M25.51, M25.55 - M25.57, M43.2 - M43.6, M45, M46.1, M46.3, M46.4, M46.9, M47, M48.0, M48.1, M48.8, M48.9, M50.8, M50.9, M51, M53.1 - M53.3, M53.8, M53.9, M54, M60.8, M60.9, M63.3, M79.0 - M79.2, M79.6, M79.7, M96.1 | No |
| Chronic pulmonary disease | I27.8, I27.9, J40–J44, J46-J47, J60–J67, J68.4, J70.1, J70.3 | Yes |
| Chronic viral hepatitis B | B16, B18.0-B18.1 (2 hospitalisations) | No (but, “Abnormal liver function tests (in the absence of an underlying cause)”) |
|  | K70.3, K74.3, K74.4, K74.5, K74.6 (1 hospitalisation) |  |
| Cirrhosis | I85.0, I85.9, I98.2, I98.3, K65.0, K65.8, K65.9, K67.0, K67.1, K67.2, K67.3, K67.8, K76.7, K93.0, R18 | No (but, “Abnormal liver function tests (in the absence of an underlying cause)”) |
| Dementia | F00–F03, F05.1, G30, G31.1 | Yes |
| Depression | F20.4, F31.3–F31.5, F32, F33, F34.1, F41.2, F43.2 | Yes |
| Diabetes | E10-E14 | Yes |
| Epilepsy | G40-G41 | Yes |
| Hypertension | I10-I13, I15 | Yes |
| Hypothyroidism | E00–E03, E89.0 | No |
| Inflammatory bowel disease | K50, K51 | No |
| Irritable bowel syndrome  (2 hospitalisations) | K58 | No |
|  | Exclude C18-C21, C25, C56, C78.5, C79.6, D01.7, D01.9, D37.1-D37.5, K50-K51, K70.2-K70.3, K74.0, K74.2, K74.6, K86.0-K86.1, K90, K91.2 |  |
| Multiple sclerosis  (2 hospitalisations) | G35, G36, G37, H46 | Yes |
| Myocardial infarction | I21-I22 | Yes |
| Parkinson’s disease | G20, G21, G22 | No |
| Peptic ulcer disease | K25.7, K25.9, K26.7, K26.9, K27.7, K27.9, K28.7, K28.9 | No |
| Peripheral vascular disease | I70.2 | No |
| Psoriasis | L40.0 - L40.4, L40.8, L40.9 | No |
| Rheumatoid arthritis | M05, M06, M31.5, M32–M34, M35.1, M35.3, M36.0 | Yes |
| Schizophrenia | F20, F21, F23.2, F25 | Yes |
| Severe constipation | K55.8, K56.0, K56.4, K56.7, K59.0, K63.1, K63.4, K63.81, K63.88, K92.80, K92.88 | No |
|  |  |  |
|  | Exclude C17-C21, C45.1, C48, C51-C58, C60-C68, C78.5-C78.6, D01.7, D01.9, D37.1-D37.5, K50-K51, K66.0, N73.6, N99.4 (K56.6 if R10.1), and any CCPx surgery listed in claims |  |
| Stroke or TIA | G45.0-G45.3, G45.8-G45.9, H34.1, I60, I61, I63, I64 | Yes |
