# Supplementary material for: Multimorbidity combinations, costs of hospital care and potentially preventable emergency admissions in England: A cohort study
Source: PLoS Med. 2021 Jan 13;18(1):e1003514. doi: 10.1371/journal.pmed.1003514 (PMC7815339; doi:10.1371/journal.pmed.1003514)
Supplement: S3 Appendix — (DOCX) [file pmed.1003514.s003.docx]

# S3 Appendix. Cohort sample coding

We include all patients in the cohort with an inpatient elective or emergency admission, as defined in the Hospital Episode Statistics Data Dictionary for Admitted Patient Care (<https://digital.nhs.uk/data-and-information/data-tools-and-services/data-services/hospital-episode-statistics/hospital-episode-statistics-data-dictionary>).

Emergency admission ‘admimeth’ codes included: 21, 22, 23, 24, 28, 2A, 2B, 2C, 2D

Elective admission ‘admimeth’ codes included: 11, 12, 13

Authors had access to the entire HES admitted care, outpatient and A&E datasets for the 2009/10 to 2017/18 period to construct the study cohort. Access to HES data is available via a data sharing agreement with NHS Digital.
