# Supplementary material for: Multimorbidity combinations, costs of hospital care and potentially preventable emergency admissions in England: A cohort study
Source: PLoS Med. 2021 Jan 13;18(1):e1003514. doi: 10.1371/journal.pmed.1003514 (PMC7815339; doi:10.1371/journal.pmed.1003514)
Supplement: S4 Appendix — (DOCX) [file pmed.1003514.s004.docx]

# S4 Appendix. Ambulatory care sensitive conditions – emergency admissions

Emergency admission for ACSC (from list below) as primary diagnosed condition (Adapted from Harrison Mark J, Dusheiko Mark, Sutton Matt, Gravelle Hugh, Doran Tim, Roland Martin. Effect of a national primary care pay for performance scheme on emergency hospital admissions for ambulatory care sensitive conditions: controlled longitudinal study *BMJ* 2014; 349 :g6423)

|  |  |
| --- | --- |
| **ACSC condition** | **ICD-10** |
| Asthma | J45, J46 |
| Chronic Ischaemic Heart Disease | I20, I240, I248, I249, I25 |
| Congestive Heart Failure | I110, I130, I50, J81 |
| COPD | J20, J41, J42, J43, J44, J47 |
| Diabetes | E100-108, E110-118, E130-138, E140-148 |
| Epilepsy and convulsions | G40, G41, G568, R568 |
| Hypertensions | I10, I119 |
| Diabetes Hypo | E162 |
| Iron-deficiency anaemia | D501, D508, D509 |
| Cellulitis | L03, L04, L080, L088, L089, L88, L980 |
| Dehydration & gastroenteritis | E86, K522, K528, K529 |
| ENT | H66, H67, J02, J03, J04, J06, J312 |
| Gangrene | R02 |
| Nutritional deficiency | E40, E41, E42, E43, E550, E643 |
| Pelvic inflammatory | N70, N73, N74 |
| Perforated/bleeding ulcer | K250-256, K260-262, K264-266, K270-K272, K273-276, K280-282, K284-286 |
| Polynephritis and UTI | N10, N11, N12, N136, N300, N308, N309 |
| Other vaccine preventable diseases | A35, A36, A37, A80, B05, B06, B161, B169, B180, B181, B26, G000, M014 |
