# Supplementary material for: Multimorbidity combinations, costs of hospital care and potentially preventable emergency admissions in England: A cohort study
Source: PLoS Med. 2021 Jan 13;18(1):e1003514. doi: 10.1371/journal.pmed.1003514 (PMC7815339; doi:10.1371/journal.pmed.1003514)
Supplement: S5 Appendix — (DOCX) [file pmed.1003514.s005.docx]

# S5 Appendix. Distribution of costs per unique multimorbidity (MM) combination

|  | Total costs of secondary care | Potentially preventable (ACSC) costs |
| --- | --- | --- |
| 2017/18 costs | 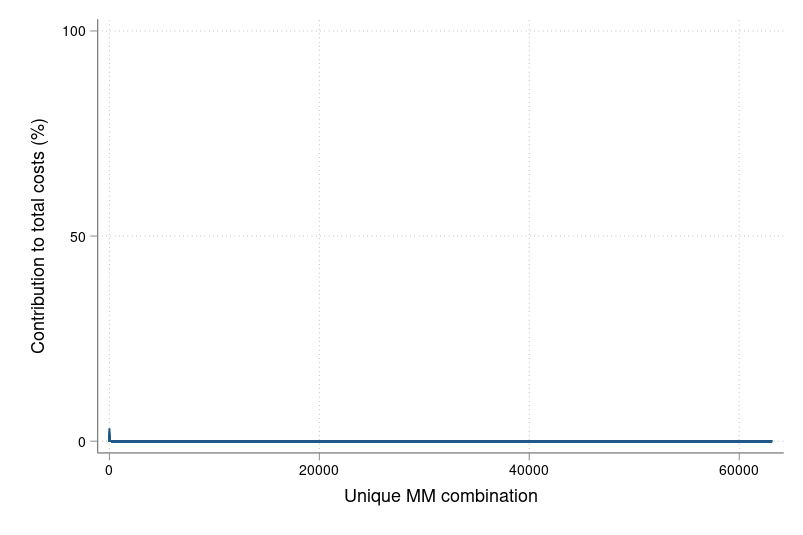 | 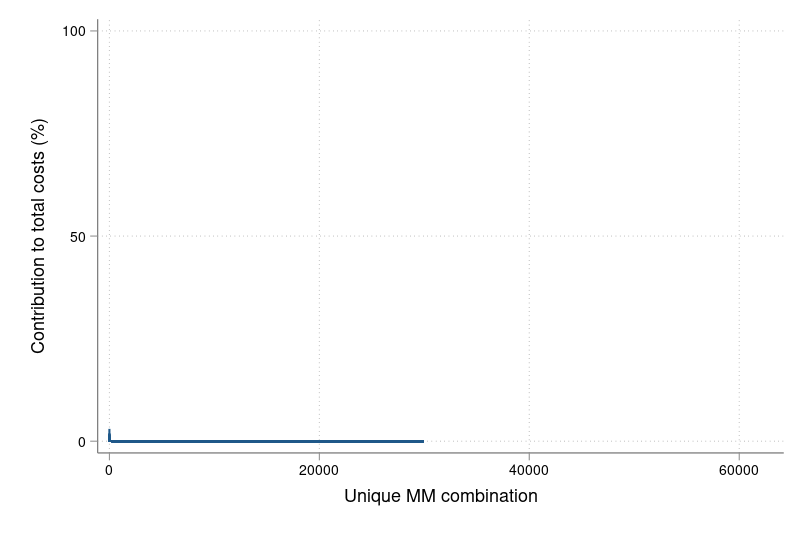 |
| 5-year costs | 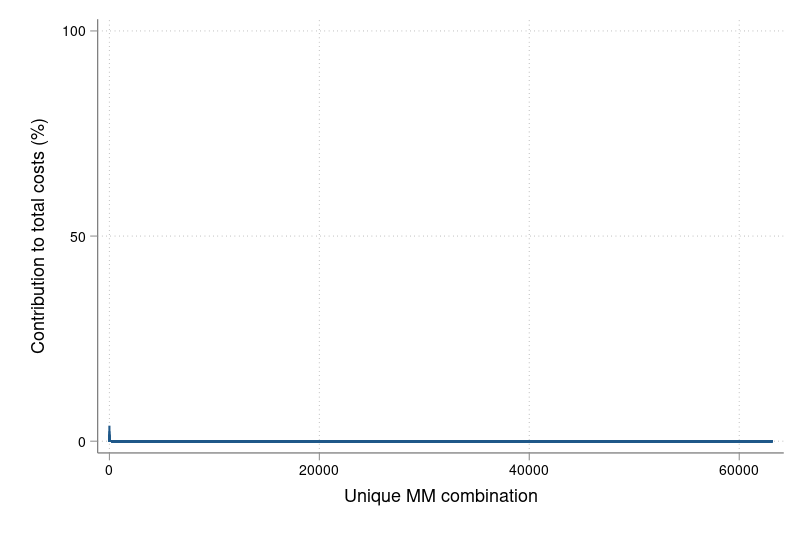 | 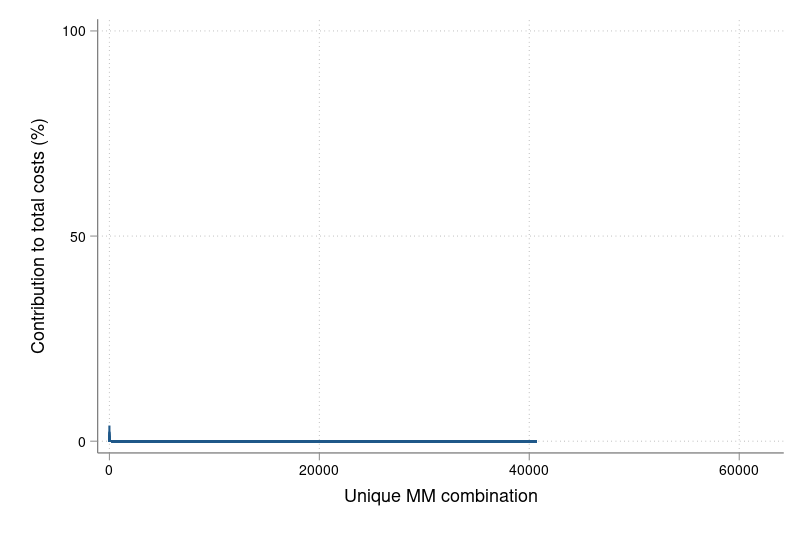 |
