# Supplementary material for: Multimorbidity combinations, costs of hospital care and potentially preventable emergency admissions in England: A cohort study
Source: PLoS Med. 2021 Jan 13;18(1):e1003514. doi: 10.1371/journal.pmed.1003514 (PMC7815339; doi:10.1371/journal.pmed.1003514)
Supplement: S6 Appendix — (DOCX) [file pmed.1003514.s006.docx]

# S6 Appendix. Top ten combinations by age and deprivation

Over 65s

| Rank | Conditions in combination (count) | Rank in total MM population | Percent of total cost for over65 MM patients (%) | Total cost of secondary care (£m) | Count of unique patients with combination |
| --- | --- | --- | --- | --- | --- |
| 1. | Diabetes, hypertension  (2) | 1 | 3.04 | £301.66 | 109,955 |
| 2. | Kidney, hypertension  (2) | 2 | 2.57 | £254.89 | 55,109 |
| 3. | Cancer, hypertension (2) | 3 | 1.83 | £181.45 | 34,232 |
| 4. | Kidney, diabetes, hypertension  (3) | 4 | 1.64 | £162.37 | 31,029 |
| 5. | Pulmonary, hypertension  (2) | 5 | 1.46 | £144.74 | 42,910 |
| 6. | Pain, hypertension  (2) | 6 | 1.19 | £118.39 | 35,510 |
| 7. | CHF, Kidney, hypertension  (3) | 9 | 1.04 | £103.26 | 13,747 |
| 8. | Asthma, hypertension (2) | 7 | 1.00 | £98.70 | 36,598 |
| 9. | Hypertension, hypothyroidism  (2) | 8 | 0.94 | £93.20 | 28,872 |
| 10. | CHF, hypertension  (2) | 10 | 0.87 | £86.16 | 17,948 |

Under 65s

| Rank | Conditions in combination (count) | Rank in total MM population | Percent of total cost for under65 MM patients (%) | Total cost of secondary care (£m) | Count of unique patients with combination |
| --- | --- | --- | --- | --- | --- |
| 1. | Diabetes, hypertension  (2) | 1 | 3.549 | £150.61 | 58,885 |
| 2. | Kidney, hypertension  (2) | 2 | 1.93 | £83.36 | 17,649 |
| 3. | Asthma, depression  (2) | 18 | 1.50 | £64.60 | 25,313 |
| 4. | Asthma, hypertension (2) | 7 | 1.45 | £62.63 | 25,747 |
| 5. | Kidney, diabetes, hypertension  (3) | 4 | 1.39 | £60.16 | 10,085 |
| 6. | Cancer, hypertension (2) | 3 | 1.39 | £59.78 | 10,084 |
| 7. | Pain, hypertension  (2) | 6 | 1.24 | £53.49 | 18,813 |
| 8. | Asthma, pain  (2) | 21 | 1.23 | £54.20 | 19,775 |
| 9. | Pain, depression  (2) | 25 | 1.20 | £51.81 | 17,510 |
| 10. | Alcohol, depression  (2) | 30 | 1.12 | £48.18 | 22,662 |

IMD decile 1 (wealthiest)

| Rank | Conditions in combination (count) | Rank in total MM population | Percent of total cost for IMD1 MM patients (%) | Total cost of secondary care (£m) | Count of unique patients with combination |
| --- | --- | --- | --- | --- | --- |
| 1. | Diabetes, hypertension  (2) | 1 | 3.17 | £40.70 | 14,796 |
| 2. | Kidney, hypertension  (2) | 2 | 2.87 | £36.90 | 8066 |
| 3. | Cancer, hypertension (2) | 3 | 2.50 | £32.11 | 5830 |
| 4. | Pain, hypertension  (2) | 6 | 1.53 | £19.66 | 6125 |
| 5. | Kidney, diabetes, hypertension  (3) | 4 | 1.42 | £18.20 | 3330 |
| 6. | Asthma, hypertension (2) | 7 | 1.39 | £17.86 | 6906 |
| 7. | Hypertension, hypothyroidism  (2) | 8 | 1.11 | £14.28 | 4490 |
| 8. | Pulmonary, hypertension  (2) | 5 | 1.05 | £13.48 | 3904 |
| 9. | CHF, Kidney, hypertension  (3) | 9 | 1.01 | £12.92 | 1658 |
| 10. | CHF, hypertension  (2) | 10 | 0.97 | £12.47 | 2537 |

IMD decile 10 (most deprived)

| Rank | Conditions in combination (count) | Rank in total MM population | Percent of total cost for IMD10 MM patients (%) | Total cost of secondary care (£m) | Count of unique patients with combination |
| --- | --- | --- | --- | --- | --- |
| 1. | Diabetes, hypertension  (2) | 1 | 2.95 | £43.93 | 16,838 |
| 2. | Kidney, hypertension  (2) | 2 | 1.82 | £27.11 | 5550 |
| 3. | Kidney, diabetes, hypertension  (3) | 4 | 1.57 | £23.33 | 4295 |
| 4. | Pulmonary, hypertension  (2) | 5 | 1.47 | £21.90 | 6732 |
| 5. | Cancer, hypertension (2) | 3 | 0.94 | £13.96 | 2471 |
| 6. | Pain, hypertension  (2) | 6 | 0.84 | £12.47 | 4193 |
| 7. | Asthma, hypertension (2) | 7 | 0.80 | £11.96 | 4982 |
| 8. | Chronic heart failure, chronic kidney disease, diabetes, hypertension  (4) | 11 | 0.75 | £11.16 | 1205 |
| 9. | Asthma, depression  (2) | 18 | 0.72 | £10.73 | 4158 |
| 10. | Alcohol, depression  (2) | 30 | 0.63 | £9.40 | 4029 |
