# Supplementary material for: Multimorbidity combinations, costs of hospital care and potentially preventable emergency admissions in England: A cohort study
Source: PLoS Med. 2021 Jan 13;18(1):e1003514. doi: 10.1371/journal.pmed.1003514 (PMC7815339; doi:10.1371/journal.pmed.1003514)
Supplement: S7 Appendix — (DOCX) [file pmed.1003514.s007.docx]

# S7 Appendix. Distribution and top ten combinations, dropping hypertension

|  | Total costs of secondary care | Potentially preventable (ACSC) costs |
| --- | --- | --- |
| 2017/18 costs | 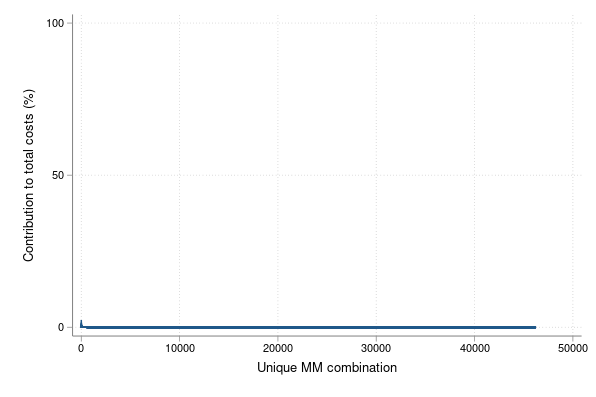 | 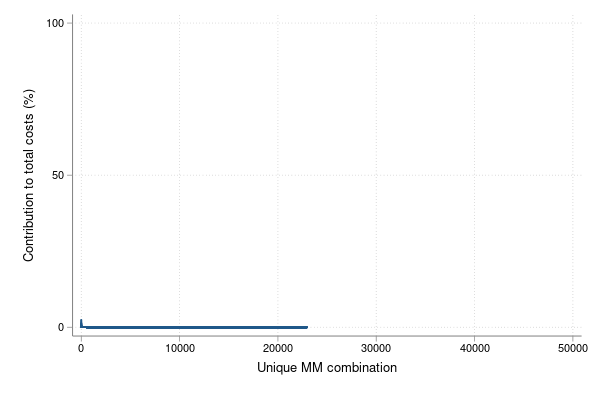 |
| 5-year costs | 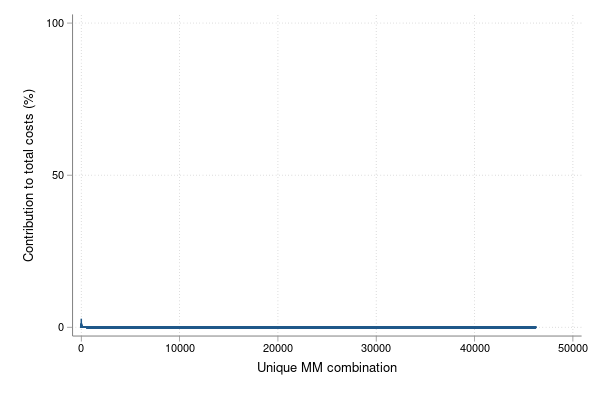 | 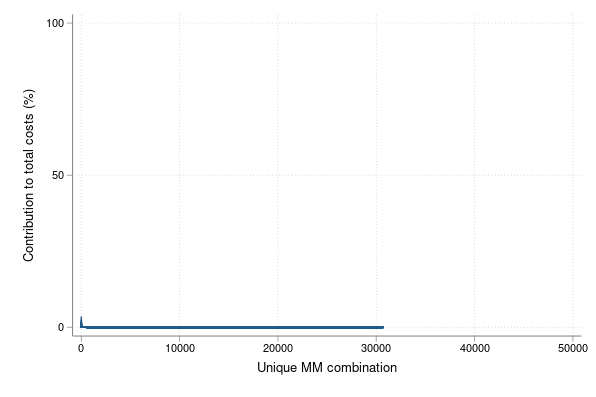 |

| Rank | Conditions in combination (count) | Percent of total cost for MM patients (%) | Percent of total cost for all patients (%) | Total cost of secondary care (£m) | Count of unique patients with combination |
| --- | --- | --- | --- | --- | --- |
| 1. | Kidney, diabetes  (2) | 2.41 | 1.12 | £292.46 | 58,595 |
| 2. | Cancer, kidney  (2) | 1.59 | 0.73 | £192.03 | 22,798 |
| 3. | CHF, kidney (2) | 1.39 | 0.64 | £168.73 | 23,456 |
| 4. | CHF, kidney, diabetes  (3) | 1.07 | 0.49 | £129.31 | 15,474 |
| 5. | Cancer, diabetes  (2) | 0.94 | 0.43 | £113.26 | 20,491 |
| 6. | Kidney, pulmonary  (2) | 0.93 | 0.43 | £112.76 | 20,508 |
| 7. | Cancer, pulmonary  (3) | 0.89 | 0.41 | £107.60 | 17,364 |
| 8. | Kidney, dementia (2) | 0.79 | 0.37 | £96.23 | 17,343 |
| 9. | Pulmonary, diabetes  (2) | 0.78 | 0.36 | £93.94 | 28,362 |
| 10. | Asthma, diabetes  (2) | 0.77 | 0.36 | £93.52 | 35,931 |
