# Supplementary material for: Multimorbidity combinations, costs of hospital care and potentially preventable emergency admissions in England: A cohort study
Source: PLoS Med. 2021 Jan 13;18(1):e1003514. doi: 10.1371/journal.pmed.1003514 (PMC7815339; doi:10.1371/journal.pmed.1003514)
Supplement: S8 Appendix — (DOCX) [file pmed.1003514.s008.docx]

# S8 Appendix. Distribution and top ten combinations, only 15 NHS guidance conditions

|  | Total costs of secondary care | Potentially preventable (ACSC) costs |
| --- | --- | --- |
| 2017/18 costs | 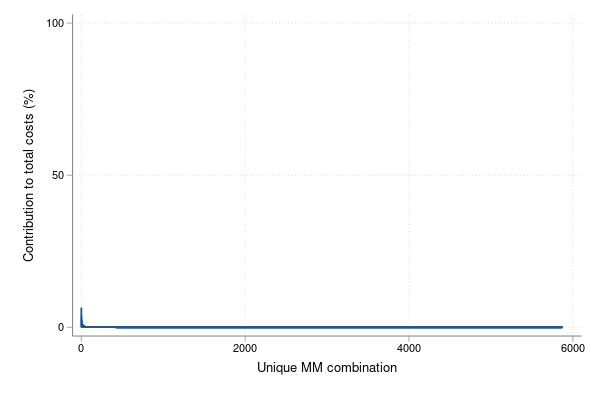 | 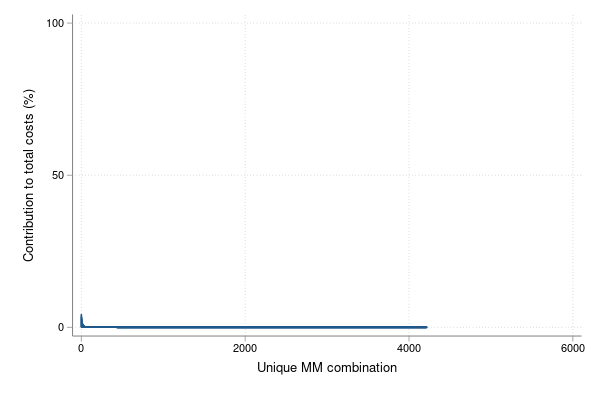 |
| 5-year costs | 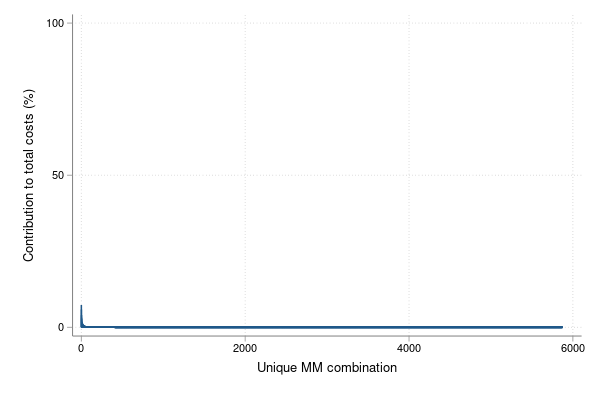 | 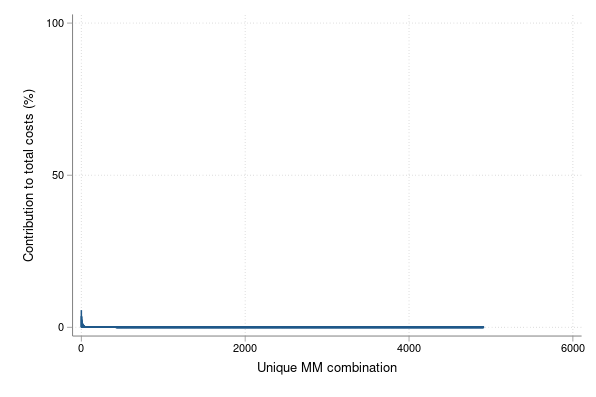 |

| Rank | Conditions in combination (count) | Percent of total cost for MM patients (%) | Percent of total cost for all patients (%) | Total cost of secondary care (£m) | Count of unique patients with combination |
| --- | --- | --- | --- | --- | --- |
| 1. | Diabetes, hypertension  (2) | 6.49 | 2.89 | £757.20 | 239,804 |
| 2. | Kidney, hypertension  (2) | 6.29 | 2.80 | £734.44 | 127,380 |
| 3. | Kidney, diabetes, hypertension (3) | 3.90 | 1.73 | £454.80 | 70,384 |
| 4. | Pulmonary, hypertension  (2) | 2.88 | 1.28 | £335.73 | 81,623 |
| 5. | Asthma, hypertension  (2) | 2.37 | 1.06 | £277.03 | 91,305 |
| 6. | CHF, kidney, hypertension  (3) | 2.16 | 0.96 | £252.23 | 28,228 |
| 7. | CHF, kidney, diabetes, hypertension  (4) | 1.88 | 0.84 | £219.79 | 21,455 |
| 8. | CHF, hypertension (2) | 1.64 | 0.73 | £191.93 | 34,345 |
| 9. | Depression, hypertension  (2) | 1.50 | 0.67 | £174.81 | 45,538 |
| 10. | Kidney, pulmonary, hypertension  (3) | 1.34 | 0.60 | £156.90 | 21,821 |
