# Supplementary material for: Multimorbidity combinations, costs of hospital care and potentially preventable emergency admissions in England: A cohort study
Source: PLoS Med. 2021 Jan 13;18(1):e1003514. doi: 10.1371/journal.pmed.1003514 (PMC7815339; doi:10.1371/journal.pmed.1003514)
Supplement: Attachment — Submitted filename: Reply to reviewers_vFINAL.docx [file pmed.1003514.s009.docx]

Dear Editor,

Thank you to you and the reviewers for providing very helpful comments on our previous submission. We appreciate the opportunity to revise the article and feel the comments have enabled us to improve the attached revisions substantially.

Please find below our point-by-point responses and attached the corresponding tracked changes to the manuscript. All page references below refer to the clean version of the manuscript, without tracked changes.

We look forward to your decision.

Best wishes,

Jonathan Stokes, Bruce Guthrie, Stewart W Mercer, Nigel Rice, Matt Sutton

Requests from the editors:

*At this stage, we ask that you include a short, non-technical Author Summary of your research to make findings accessible to a wide audience that includes both scientists and non-scientists. The Author Summary should immediately follow the Abstract in your revised manuscript. This text is subject to editorial change and should be distinct from the scientific abstract. Please see our author guidelines for more information: <https://journals.plos.org/plosmedicine/s/revising-your-manuscript#loc-author-summary>

- RESPONSE: We have added an author summary.
- (p.4) “**Why Was This Study Done?**
- Multimorbidity, the presence of two or more chronic conditions in an individual, results in worse outcomes for patients and higher costs to health systems.
- Because existing interventions show little success, researchers have begun looking for specific combinations, ‘clusters’, of conditions that might be treated more effectively.
- This existing analysis has focused on combinations of conditions that are common in the general population, but these may not be the combinations that are associated with the most important outcomes from a health service perspective.
- **What Did the Researchers Do and Find?**
- We used national hospital data to realign research into multimorbidity by starting with outcomes relevant to the healthcare system, total costs of secondary care and costs of (potentially preventable) emergency admissions.
- We identified all unique combinations of 28 conditions present in a cohort of over 8 million people admitted to a hospital in England between April 2017 and March 2018.
- We examined the distribution and top contributors of total costs of these combinations over one and five years for the whole cohort and the average costs per individual.
- We additionally highlighted overlaps between unique combinations, which may merit follow-up in terms of development of further disease and costs.
- We identified over 60,000 unique disease combinations. No combination accounted for more than 3.2% of total costs for multimorbid patients. The combinations with the highest average cost per patient were not the same as those with highest total cost.
- **What Do These Findings Mean?**
- There are no clear discrete disease combinations at which to target interventions, which implies a generalist/multidisciplinary team approach will remain important rather than pathways/guidelines based on a few specific disease clusters.
- Combinations containing the highest cost patients (the current focus of many interventions) were different to those accounting for the highest total costs, implying the need to develop interventions beyond only high-risk patients.
- There might be scope to use clusters to understand and develop preventative interventions, but focusing on addressing well-known disease risk factors (such as obesity, diet, exercise and deprivation) with public health/primary care interventions might provide the most efficient route to benefit systems financially and benefit many multimorbid patients.”

  *In the last sentence of the Abstract Methods and Findings section, please include a brief note about any key limitation(s) of the study's methodology.
- RESPONSE: We have added a brief note on key limitations to the abstract.
- (p.2) “Key limitations include availability of data only from hospitals and reliance on hospital coding of health conditions.”

*Please clarify if the analytical approach reported here corresponds to one laid out in a prospective protocol or analysis plan? Please state this (either way) early in the Methods section.

- RESPONSE: Added clarifying statement to first section of methods.
- (p.6) “This study is based on exploratory analysis of data as part of a research Fellowship on health economics of multimorbidity (MRC Grant Ref: MR/T027517/1), but not based on a specific prospective analysis plan.”

a) If a prospective analysis plan (from your funding proposal, IRB or other ethics committee submission, study protocol, or other planning document written before analyzing the data) was used in designing the study, please include the relevant prospectively written document with your revised manuscript as a Supporting Information file to be published alongside your study, and cite it in the Methods section. A legend for this file should be included at the end of your manuscript. 

b) If no such document exists, please make sure that the Methods section transparently describes when analyses were planned, and when/why any data-driven changes to analyses took place.

- RESPONSE: Added a statement on when analyses were planned. No data-driven changes to analyses applicable. Below we have also included details of the additional sensitivity analysis incorporated in response to reviewer comments.
- (p.6) “Analyses were planned in author discussions in January 2020.”

c) In either case, changes in the analysis-- including those made in response to peer review comments-- should be identified as such in the Methods section of the paper, with rationale.

- RESPONSE: We have added a summary of changes made in response to peer-review comments.
- (p.8) “Following peer-review feedback we were asked to explore the effects of dropping hypertension as a condition, as arguably it might be considered a symptom or risk-factor, and to consider the effects of only including a smaller number of conditions on the interpretation of results. We added these as sensitivity analyses, re-running the main analysis exploring the distribution of costs and top ten conditions contributing to total secondary care costs for all unique: (i) combinations of 27 conditions, excluding hypertension from our original list; (ii) combinations of 15 conditions, the 15 conditions with explicit NHS guidance on co-morbidity recording (as described above and in the Appendix).”

*It would be helpful to use an appropriate reporting guideline to support reporting of the study, and perhaps also to trigger filling in some of the key details noted by reviewers (below). The RECORD guideline (<https://www.equator-network.org/reporting-guidelines/record/>) may be most appropriate here, designed to support reporting of studies done using routinely-collected health data. If using this guideline please note that in the methods section and include a completed checklist as a supporting information file (which should then be called out in Methods).

- RESPONSE: Added completed RECORD checklist and called out in methods.
- (p.6) “The study was reported in line with the RECORD guidelines [20].”

-----------------------------------------------------------

Comments from the reviewers:

Reviewer #1: I confine my remarks to statistical aspects of this paper.

I have one question: Were the circles in the figure proportional by the area or the diameter of the circle? (It should be area).

- RESPONSE: Yes, circle size is proportional to area in figure 1. We have now clarified this in the figure description.
- (p.13) “Area of circles are weighted by proportion of total cost that is potentially preventable (ACSC), larger circle indicates higher proportion of potentially preventable costs.”

But this is a very interesting problem, statistically speaking. As the authors note, with so many conditions, there are millions and millions of possible combinations, far more than even the large number of subjects. The authors look at every combination that appeared in the data, no matter how complex. This runs the risk of overfitting the data. Although the authors did not do regression or something similar, they did look at the cost per patient of some very complex combinations. But it seems likely that many of the combinations that were not observed would have very high costs, too.

- RESPONSE: This is a very important point from a statistical perspective. However, in this paper, our main aim is to address the proposed ‘solution’ in the current multimorbidity literature of simplifying the multimorbidity problem by selecting a few prevalent clusters (or combinations) of conditions. As the reviewer rightly points out, our purpose, therefore, is to describe the observed (rather than all possible) combinations in the hospital data and their associated costs. We do this for a cohort of patients with an inpatient admission in England in 2017/18. The fact that we find so many combinations, none of which is a major driver of total system costs, is what we highlight as our main finding. As you suggest, we do not attempt to model this and risk over-fitting, the analysis is purposefully descriptive to avoid this. We have, however, added the important limitation that you highlight: that there will be combinations of conditions that we have not observed in the year we have examined.
- (p.14-15) “We also observed only a fraction of the 268 million possible condition combinations, so there are likely to be many other drivers of costs in other patients in other years. However, we do not expect the distribution of costs or costliest combinations to change drastically with the inclusion of more rare combinations.”

I think a couple of additional analyses would be very useful.

First, what if all the costs per patient were randomly assigned in some fashion?

- RESPONSE: This would be a very interesting analysis if we were to focus on how changes to the pricing/costs for each group of conditions would change the distribution. As above, however, we are focusing this paper solely on observed paid costs to comment directly on the legitimacy (in terms of this single, important, outcome) of the cluster analysis method for identifying a manageable number of conditions. We have added to the discussion section.
- (p.16) “The effects of current payment tariffs on multimorbidity costs and differences across condition combinations also merits further work.”

Second, the authors could look at all unique dyads and triads of conditions. With 28 conditions there are 28*27/2 = 378 dyads and 9828 triads.  This suits the sample size.

- RESPONSE: We agree that observing dyads/triads would simplify the analysis. However, we feel it risks over-simplifying for the purpose of this study - something which we highlight as a weakness in the existing literature. Individually, those with more conditions are known to be the most complex to treat and tend to be higher cost, as we illustrate in our analysis of costs per patient. Therefore, we could not clinically justify a focus only on those with fewer conditions. Indeed, part of the intrigue of the findings we present is that these relatively simpler combinations (dyads/triads) are the highest cost at the system level (even if not at the individual patient level), even when all possible combinations are included. However, we have incorporated a sensitivity analysis with a simpler set of conditions, as outlined in response to your comment below.

Third, perhaps some of the conditions could be combined? This is more a medical question than a statistical one, so I can't really suggest combinations. But, as an educated layperson, it seems to me that there should be some combination possible. With 10 "meta-conditions" there would be 2^10 -11 - 1013 combinations and this is much more manageable.  Even 15 conditions wouldn't be too bad.

- RESPONSE: We agree, examining the distribution with a smaller combination of possible conditions is an interesting methodological question. We now run as a sensitivity analysis using only the 15 conditions given explicit guidance on coding by the NHS (also those conditions we have most confidence in being correctly recorded, as recommended also by reviewer 3).
- (p.8) “Following peer-review feedback we were asked to explore the effects of dropping hypertension as a condition, as arguably it might be considered a symptom or risk-factor, and to consider the effects of only including a smaller number of conditions on the interpretation of results. We added these as sensitivity analyses, re-running the main analysis exploring the distribution of costs and top ten conditions contributing to total secondary care costs for all unique: (i) combinations of 27 conditions, excluding hypertension from our original list; (ii) combinations of 15 conditions, the 15 conditions with explicit NHS guidance on co-morbidity recording (as described above and in the Appendix).”
- (p.13) “Both sensitivity analyses gave broadly the same results as above, no clear cost driving combinations, and those combinations with highest cost those with fewer conditions but affecting a larger proportion of the population (see Appendix). When dropping hypertension, the highest cost combination was chronic kidney disease and diabetes, contributing to 2.4% of total secondary care costs for multimorbid patients, 1.1% of costs for all patients. When analysing only 15 conditions, there were by design far fewer unique combinations, 5,869 observed instead of 63,124. This inflated the proportions of total costs slightly, with more of the population assigned to each category. The highest cost combination was, like the main results, diabetes and hypertension, now contributing to 6.5% of total secondary care costs for multimorbid patients, but still only 2.9% of costs for all patients.”

Peter Flom

-----------------------------------------------------------

Reviewer #2: REVIEW of "Multimorbidity combinations, costs of hospital care and potentially preventable emergency admissions: Cross-sectional analysis of a national dataset of over 8 million people".

The article provides interesting insights into the usefulness, limits, and potential of the cluster analysis approach to healthcare usage, has a very large sample. However, the paper need further improvement efforts to be published:

-Title

Please state that your study's population is not general national population, but hospitalized patients population

- RESPONSE: We have added this clarity to the title.
- (p.1) “Multimorbidity combinations, costs of hospital care and potentially preventable emergency admissions: Retrospective cohort analysis of a national dataset of over 8 million hospitalised people”

-Methods
Please give some context information about UK NHS

- RESPONSE: We have added some brief contextual information to the methods.
- (p.6-7) “The NHS is a Beveridge healthcare system, which is tax-funded and with treatment provided free to patients at the point of delivery. NHS hospitals are predominantly paid by activity, according to a national tariff with Healthcare Resource Groups determining the prices for services [21]. NHS hospitals treat the vast majority of patients in England, with private providers accounting for, for example, just 1.3% of elective activity [22].”

If you collected outcome into five years, this is not a cross sectional design but a retrospective cohort design

- RESPONSE: Yes, a difficult classification since the design is not standard. We agree with the reviewer that there is arguably some sort of retrospective cohort aspect since we define our participants cross-sectionally and subsequently trace their hospital usage and costs across the previous five years. We have amended the title to reflect this.
- (p.1) “Multimorbidity combinations, costs of hospital care and potentially preventable emergency admissions: Retrospective cohort analysis of a national dataset of over 8 million hospitalised people”

Please explain how do you count for the five years costs and which time period do you analyze.

- RESPONSE: We first constructed our cohort on all patients with an inpatient admission in the 2017/18 financial year, i.e. April 2017 to March 2018. We subsequently added the relevant patients’ hospital activity data over the previous five years (i.e. 2013/14-2017/18) and summed the costs assigned to the yearly activity using the national tariffs that applied in each year. We have added additional details to clarify.
- (p.7) “Five-year costs are additionally summed for each patient over 2013/14-2017/18, again based on the hospital activity data linked to the applicable national tariff for each year.”

Please provide a unique subheading "statistical analysis" grouping the actual subheadings "analysis", "targeting intervention", "targeting prevention"….

- RESPONSE: We have amended the sub-heading as the reviewer advised.

(p.7) “Statistical analysis”

First phrase of methods could be moved into the discussion.

- RESPONSE: We have decided to keep this information in the methods section, as it provides the necessary reasoning for the additional sensitivity analysis using the 15 conditions which the NHS provides specific guidance on (see response to reviewers 1 and 3). However, we have moved this to the new ‘context’ section, which we added in response to your comment above.
- (p.7) “Our ability to record multimorbidity in this setting relies on good quality data recording multiple co-morbidities for each patient. Accuracy of diagnosis recording has improved in the hospital setting in England since it was linked to payments in 2002. A systematic review in 2011 found median diagnostic recording accuracy of 80%, up to 96% for the primary condition [23]. Additionally, NHS guidance since 2010 has stated that “any co-morbidity that affects the management of the patient and contributes to an accurate clinical picture within the current episode of care must be recorded” and lists 61 co-morbidities that must always be recorded regardless (15 of the 28 conditions we record in this study are on this list – see Appendix) [24].”

The part of analyses, which you define as sensitivity analyses, could be better defined as analyses of subgroups, please  revised in the text

- RESPONSE: Revised.
- (p.8) “Subgroup analysis”

-Results

There's no mentioning of the prevalence of the most common conditions among your population sample. I suggest you to provide a table one with the description of sample characteristics and the prevalence of the most common conditions among your sample (regardless of combinations) to understand how it is representative on entire population. 
You can create a figure with two columns representing in one the distribution of number of conditions in the sample (e.g. 42,4% no condition…) and in other the distribution of total costs by condition group (23.3%  of cost for no condition)

- RESPONSE: Thank you for the suggestion. We have now added this table.
- (p.9-10) “Table 2 shows the sample characteristics by individual condition. Hypertension was the most prevalent morbidity recorded in the sample, recorded in over a quarter of patients (26.5%). Diabetes (11.6%), chronic kidney disease (10.3%) and asthma (9.5%) were next most common, recorded in a tenth of the sample. In terms of costs, patients with hypertension contributed to 41.3% of total costs of secondary care, followed by chronic kidney disease (24.3%), both higher than the total contribution of those with no conditions.”

In table 1 is lacking sometime of labels of the outputs (for example age will be reported mean and standard deviation). Moreover, please provide percentages out of bracket and absolute numbers inside. Please provide both currencies reported in the labels. Why there are so many with missing age it not compulsory in health care records to add date of birth?

- RESPONSE: We have updated the formatting and labelling of table 1, as advised. We have also double-checked our dataset, and there are indeed missing age data. We have also checked the wider literature to confirm. A study by Herbert et al. 2017 (<https://academic.oup.com/ije/article/46/4/1093/3072145>), for example, recorded 0.5% missing finished consultant episodes in 2014/15, which we can replicate in our data. This corresponds to a rate of 1.2% when the denominator is aggregated in terms of unique patients. There are more episodes recorded in total and also slightly more missing in the most recent data we have available, the 2017/18 data we use to construct our cohort, with 1.24% missing finished consultant episodes. However, on checking our date we also spotted a small coding error that led to 183,951 observations aged <1 year (coded 7001-7009 in the HES data) having been mistakenly re-coded as missing. We have corrected this error, which reduces the amount of missingness in terms of individual patients from 4.75% to 2.57% of the sample. We only use the age variable when we break down the results by over/under 65 in subgroup analysis (now also updated), so we keep these persons with missing age in the sample for the main analysis despite being dropped from the subgroups.
- (p.8) “Those with missing age or deprivation are excluded from the subgroup analyses, but included in the main analyses which does not require these variables.”
- (p.9) “Percent of sample (n) […] Age, mean (SD)”

In table 2 do you report only in pounds? You refer the labels of columns as "total costs", but do they refer to "average total costs"?

- RESPONSE: Yes, you’re correct, in table 2 (now table 3) the monetary units are always pounds, also columns of percentage of this total cost, where labelled. The columns correctly refer to “total costs”, i.e. summed across all patients with those conditions over the period of time indicated since we are interested in total system cost impact here. Table 3 (now table 4), per patient costs, are averages, though, which we have now made clearer.
- (p.12) “Average annual cost of secondary care per patient (£)”

In table 1 and 2 please provide how frequent ( prevalence of combinations) are the combinations in the MM patients

- RESPONSE: In the first table, unfortunately there are far too many combinations to report the prevalence of all combinations. The prevalence of the combinations are reported in the tables showing the top tens, however, the column labelled “Count of unique patients with combination”. We have also added the percentage of multimorbid patients this count encompasses as additional information to these tables.
- (p.11-12) “Count of unique patients with combination (% of all MM patients)”

Please give at least one decimal in the figure of percentage reported in the text

- RESPONSE: Updated.
- (p.8) “Nearly 5m (57.6%) had at least one chronic condition, and over 2m (31.6%) had multimorbidity (two or more of the 28 recorded chronic conditions).”
- (p.11) “Only seven of the 63,124 unique combinations of co-conditions contribute any more than 1% of total cost of secondary care for multimorbid patients, with a maximum of approximately 3.2% in any one unique set (Table 3). Only two unique combinations contribute more than 1% of total cost of secondary care for all patients, max of 1.7%.”
- (p.12) “All combinations containing both chronic kidney disease + hypertension contribute nearly a third (29.1%) of total costs of secondary care for multimorbid patients (16.1% of costs for all patients). Combinations containing diabetes + hypertension contribute nearly a quarter (24.3%) of total costs of secondary care for multimorbid patients (13.5% of costs for all patients). The combinations containing chronic heart failure + hypertension, and chronic heart failure + chronic kidney disease + hypertension each have the highest proportion of total cost that is from potentially preventable admissions (both 18.0%).”

Fig.1: The chart's meaning is a bit difficult to understand at first sight. I suggest you introduce it with a more descriptive title, and elaborate its description a bit further in the main text. Also, I'd move the NB note above, as part of the figure's description. Finally, the chart's key placement is unfortunate: I suggest you move it on the top right corner and frame it, in order for the reader not to confuse it with the actual data displayed by the figure.

- RESPONSE: We agree, this could have been clearer. We have updated the title to be more descriptive. We have also updated the key placement.
- (p.13) “Figure 1: Bubble plot showing total contribution to costs of secondary care for multimorbid (MM) patients, summing costs of all multimorbid patients who have (at least) those conditions. Proportion of total costs of secondary care for multimorbid patients shown on the y-axis. Area of circles are weighted by proportion of total cost that is potentially preventable (ACSC), larger circle indicates higher proportion of potentially preventable costs.”

-Discussion
Please move the subheading "findings in the context"   before "limits"

- RESPONSE: We have moved the section, as suggested.
- (p.14) “Findings in context”

The prescribing expenditure/drugs is not on per capita basis

- RESPONSE: We can see how this sentence was not completely clear. We were referring to expenditure on primary care, that is then mostly broken down into consultation time and prescribing. We have simplified the sentence to clarify.
- (p.15) “However, arguably we are focusing on the most relevant setting in terms of NHS spending, since social care and care homes are largely paid privately by individuals with a proportion paid publicly by Local Authorities, and primary care costs on a predominantly per capita basis.”

You state "….so overall system saving are by no means guarantee….." this doubt is verified in the cost effectiveness analyses, which consider costs for each health care level. There are some evidences that found that prevention interventions even if they increase primary care costs but reducing other costs, produce net savings.

- RESPONSE: We agree there is building evidence that prevention activities, especially public health interventions, can be highly cost-effective. But, as in the case of case management interventions which try to identify high risk patients and prevent further admissions, for instance, they can also identify increased unmet need and increase costs, so savings are not guaranteed. We have now added three references on the former to better balance the argument.
- (p.15) “However, this would likely involve increased funding/staffing in other parts of the system to deliver, so overall system savings are not guaranteed. There is some evidence that, particularly public health, preventative interventions can be highly cost-effective, nevertheless [38-40].”
  - “38.	Owen L, Morgan A, Fischer A, Ellis S, Hoy A, Kelly MP. The cost-effectiveness of public health interventions. Journal of Public Health. 2011:1-9. doi: 10.1093/pubmed/fdr075.”
  - “39.	Masters R, Anwar E, Collins B, Cookson R, Capewell S. Return on investment of public health interventions: a systematic review. Journal of epidemiology and community health. 2017;71(8):827-34. doi: 10.1136/jech-2016-208141.”
  - “40.	Martin S, Lomas J, Claxton K. Is an ounce of prevention worth a pound of cure? A cross-sectional study of the impact of English public health grant on mortality and morbidity. BMJ Open. 2020;10(10):e036411. doi: 10.1136/bmjopen-2019-036411.”

-Conclusion 
"There are no clear multimorbidity combinations for a targeted intervention approach to reduce secondary care costs. The role of risk-stratification and focus on individual high-cost patients is particularly questionable." The link between these two phrases isn't very apparent. I suggest you add proper logical connectors, or re-arrange the paragraph to make it sound more consequential.

- RESPONSE: We have re-assessed the wording to better make the link.
- (p.16) “There are no clear multimorbidity combinations for a cluster-targeted intervention approach to reduce overall secondary care costs. The role of risk-stratification and focus on individual high-cost patients with interventions is particularly questionable for this aim.”

-----------------------------------------------------------

Reviewer #3: Summary
The goal of this paper is to estimate clusters of comorbidities and examine these clusters with high health care costs and avoidable hospitalizations using health administrative databases. The question and approach are interesting; however, there needs to be additional detail added to clarify the methods and outcomes. 

Comments
1. I found the points in the introduction very interesting, but they could be organized so that it is easier for the reader. I would suggest separating the analytic problems from the clinical/intervention gaps. So first clearly explain why the clusters are hard to action from an intervention perspective, and second why the methods used to identify these clusters may be limited. This study then aims to address both those gaps.

- RESPONSE: Thank you for the suggestion to make the labelling of the different issues we discuss clearer. We have now added sub-headings to the introduction and clarified in the aims that we are addressing both gaps.
- (p.5-6) “Methodological complexity… Clinical/intervention complexity”
- (p.6) “National hospital data provides the opportunity to realign research into multimorbidity by starting with outcomes of relevance to the healthcare system, rather than focusing on common sets observed in the general population. It also allows analysis of all observed disease combinations and their distribution, rather than requiring selection of only a handful.”

2. The time element of the questions was not defined. The authors pose very interesting questions regarding the relationship between different conditions with health system outcomes (costs and avoidable hospitalization), but when? Within a year? Within 30 days? It wasn't until the midpoint of the methods that it seems like it cost over five years  - but unclear form when (from the first diagnosis?). The period for when the hospitalization and costs accrual relative an index date could be more precise and justified.

- RESPONSE: We have now clarified the time-period further up in the paper, in the paper aims. As emphasised further below (response to point 4), we are most concerned with secondary care data of under-recording conditions, which means we record multimorbidity based on all available years of data. We realise this has limitations, however, especially for the analysis of five-year costs where the person might not have been multimorbid over the entire period. We have also now added this more explicitly to the limitations section.
- (p.6) “…examine the potential of defining multimorbidity clusters for impacting secondary care costs (annual, over one year, 2017/18, and previous five years, 2013/14-2017/18) via …”
- (p.15) “However, this backfilling of condition codes does not allow us to incorporate the dynamic nature of multimorbidity, i.e. that new conditions can develop over time, which means those coded as multimorbid might not have been multimorbid for the entire period (particularly the previous five-year cost analysis).”

3. Justification for the 28 conditions should be included, more than just the reference to the previous study (i.e. why were those the conditions chosen in the last study).

- RESPONSE: We have now clarified the reasoning for drawing on the coding and conditions in this previous study. The study, “Methods for identifying 30 chronic conditions: application to administrative data”, identified validated algorithms that use ICD-10 codes to ascertain the presence or absence of 40 morbidities in administrative data. The conditions were informed by a systematic review of multimorbidity measures (Diederichs C, Berger K, Bartels DB. The measurement of multiple chronic diseases–a systematic review on existing multimorbidity indices. J Gerontol A Biol Sci Med Sci. 2011;66(3):301–11.), and those used in a previous Scottish study (Barnett K, Mercer SW, Norbury M, Watt G, Wyke S, Guthrie B. Epidemiology of multimorbidity and implications for health care, research, and medical education: a cross-sectional study. Lancet. 2012;380(9836):37–43.). Of the 40 morbidities, they identified 30 that could be identified with high to moderate validity with ICD-10 codes. Three of the 30 conditions were types of cancers which we combined into a single condition category in this study.
- (p.7) “We record the presence of 28 chronic conditions according to the coding reported in a previous study which identified validated algorithms for ICD-10 coding of 30 morbidities in administrative data for the study and surveillance of multimorbidity [25]. Of the 30, three conditions were for types of cancer, which we combined into a single condition. The majority required only a single hospitalisation with a relevant ICD-10 code as any of the recorded conditions – exceptions requiring two hospitalisations were chronic pain, chronic viral hepatitis B, irritable bowel syndrome, multiple sclerosis (see Appendix).”

4. The validity of the definitions used for the 28 conditions is unclear. Many of the administrative data definitions should be validated, and those should be mentioned.  Although the authors cite diagnostic accuracy, it's unclear if they are referring to only one coding or where in the diagnostic field the code should occur (i.e. most responsible diagnosis or can be a secondary field). Generally, multiple codes/visits are needed, but it was unclear if this was done.

- RESPONSE: As above, we have now clarified the conditions of coding we used in the methods and validity of the definitions. Unfortunately, definitions for multimorbidity and coding practices in general is an under-developed research area with no commonly agreed practice, as identified in a recent Academy for Medical Sciences (AMS) Report (<https://acmedsci.ac.uk/file-download/82222577>). We do, however, follow the guidance on reporting within the AMS report, that the ICD-10 codes used should be reported explicitly (which we do in the appendix) to allow interpretation and reproducibility. In this light, we also draw on the conditions, ICD-10 codes, and reporting practices from the Tonelli et al. study, “Methods for identifying 30 chronic conditions: application to administrative data”. For this study, we are most concerned about under-reporting of conditions in administrative data (especially in the secondary care setting), which is why we backfill over multiple years of patient data. This practice is also in line with recommendations in a recent study from analysis of co-morbidity coding from US Medicare records that multiple years of data should be incorporated, where possible (<https://jamanetwork.com/journals/jamanetworkopen/fullarticle/2771115>). We have added to the limitations section also.
- (p.7) “We record the presence of 28 chronic conditions according to the coding reported in a previous study which identified validated algorithms for ICD-10 coding of 30 morbidities in administrative data for the study and surveillance of multimorbidity [25]. Of the 30, three conditions were for types of cancer, which we combined into a single condition. The majority required only a single hospitalisation with a relevant ICD-10 code as any of the recorded conditions – exceptions requiring two hospitalisations were chronic pain, chronic viral hepatitis B, irritable bowel syndrome, multiple sclerosis (see Appendix).”
- (p.15) “Unfortunately, there is no commonly agreed definition or list of ICD-10 codes for multimorbidity research [4], but we reproduce coding reported in a previously published methods paper which attempts to correct this [25]. 15 of the 28 conditions we focus on appear in the list of conditions the NHS specifically requires providers to record in this setting. Nevertheless, an audit conducted in 2014 identified consistent under-recording of co-morbidities, particularly for those whose recording is subject to clinical decision on relevancy [33]. We use all available patient data over multiple years to attempt to fill any missing data, in line with recommendations from analysis of administrative data in other settings [34], and sensitivity analysis to check the impact of more compulsory coding.”

5. Interesting that 15 conditions are given explicit guidance on coding given the NHS guidance. This, however, may introduce bias in that these conditions are more likely to be coded and therefore conditions not on this list may be missed. Can the authors comment on this?

- RESPONSE: We agree, and, as reviewer 1 also highlighted, observing fewer conditions might also influence the distributional element we are interested in. We have now included this as a sensitivity analysis. Results do not change the overall interpretation of findings.
- (p.8) “Following peer-review feedback we were asked to explore the effects of dropping hypertension as a condition, as arguably it might be considered a symptom or risk-factor, and to consider the effects of only including a smaller number of conditions on the interpretation of results. We added these as sensitivity analyses, re-running the main analysis exploring the distribution of costs and top ten conditions contributing to total secondary care costs for all unique: (i) combinations of 27 conditions, excluding hypertension from our original list; (ii) combinations of 15 conditions, the 15 conditions with explicit NHS guidance on co-morbidity recording (as described above and in the Appendix).”
- (p.13) “Both sensitivity analyses gave broadly the same results as above, no clear cost driving combinations, and those combinations with highest cost those with fewer conditions but affecting a larger proportion of the population (see Appendix). When dropping hypertension, the highest cost combination was chronic kidney disease and diabetes, contributing to 2.4% of total secondary care costs for multimorbid patients, 1.1% of costs for all patients. When analysing only 15 conditions, there were by design far fewer unique combinations, 5,869 observed instead of 63,124. This inflated the proportions of total costs slightly, with more of the population assigned to each category. The highest cost combination was, like the main results, diabetes and hypertension, now contributing to 6.5% of total secondary care costs for multimorbid patients, but still only 2.9% of costs for all patients.”

6. Hypertension is very dominant, and many argue not a condition but rather a risk factor for a chronic condition (such as diabetes or CHF). If hypertension was not included, the results would indeed be dramatically different. Can the authors comment on if this?

- RESPONSE: Yes, also a valid point. We had some early discussion amongst authors on whether to include hypertension in the main analysis but decided to include as it is commonly used in multimorbidity studies. As above, however, we have now included a sensitivity analysis where we drop hypertension from the main analysis. Results do not change the overall interpretation of findings.
- (p.8) “Following peer-review feedback we were asked to explore the effects of dropping hypertension as a condition, as arguably it might be considered a symptom or risk-factor, and to consider the effects of only including a smaller number of conditions on the interpretation of results. We added these as sensitivity analyses, re-running the main analysis exploring the distribution of costs and top ten conditions contributing to total secondary care costs for all unique: (i) combinations of 27 conditions, excluding hypertension from our original list; (ii) combinations of 15 conditions, the 15 conditions with explicit NHS guidance on co-morbidity recording (as described above and in the Appendix).”
- (p.13) “Both sensitivity analyses gave broadly the same results as above, no clear cost driving combinations, and those combinations with highest cost those with fewer conditions but affecting a larger proportion of the population (see Appendix). When dropping hypertension, the highest cost combination was chronic kidney disease and diabetes, contributing to 2.4% of total secondary care costs for multimorbid patients, 1.1% of costs for all patients. When analysing only 15 conditions, there were by design far fewer unique combinations, 5,869 observed instead of 63,124. This inflated the proportions of total costs slightly, with more of the population assigned to each category. The highest cost combination was, like the main results, diabetes and hypertension, now contributing to 6.5% of total secondary care costs for multimorbid patients, but still only 2.9% of costs for all patients.”

7. I agree overall with the interpretation that focusing on specific combinations is not likely to yield a specific path; however, I think that this does not necessarily lead to a generalist approach mentioned in the Discussion. Instead, multidisciplinary health care teams may be an equally beneficial approach - given a different mix of specialists depending on the patient's needs.

- RESPONSE: Yes, this is a good point. You’re right that this is a popular option in practice for new models of care, we have added to the text.
- (p.15) “Our results indicate no clear target combination(s) of diseases for intervention in terms of impacts on secondary care costs. The plurality likely indicates that a generalist or multidisciplinary team approach to management will remain important, rather than pathways/guidelines based on specific clusters.”
